# Longevity and Disability

**Published:** 1924-09

**Authors:** 


THE
### HOSPITAL and HEALTH REVIEW

### LONGEVITY AND DISABILITY.

Since the war came to an end few subjects have been more constantly discussed than the condition of the public health, and during the past month it has been before us even more immediately than usual. An American Professor of Psychology has assured the British Association at its Toronto meeting that the English people, hand in hand with their distant American cousins, are "speeding daily down the path that leads to destruction," and that two hundred years hence the deterioration of both of them will be complete. To him has entered Sir William Beveridge with the blunt declaration that there is no justification for the "exaggerated pessimism of eugenic propagandists," while Dr. F. C. Shrubsall, the President of the Anthropological Section, has reinforced him with comforting arguments. No sooner have the captains and the kings of science departed from Toronto than Sir George Newman steps in with his annual report, "On the State of the Public Health," with its pages of cold correctives in the shape of figures and statistics. It is an exceedingly valuable document alike as a repertory of facts and a reservoir of sane conclusions. The Chief Medical Officer reminds us that, as was already known in outline from the quarterly returns, 1923 was a "record" year. Both the general death-rate and the infant mortality rate were the lowest ever known. Every successive census shows not only that we live longer, but that longevity is increasing rapidly. In the two generations between 1841 and 1911 the number of living persons between 75 and 85 years of age increased only at the rate of 62 per hundred thousand; in the decade between 1911 and 1921 it increased by 253 per hundred thousand. The proportion of persons over 65 is now 36 per cent. greater than in 1841; on the other hand the proportion of young children is only 66 per cent. of what it was eighty years ago. We are thus well on the way to becoming a nation of the middle-aged and elderly.

The legitimate satisfaction of the sanitarian at the increasing length of life and the falling death-rate is, however, not unmitigated. Forty per cent. of last year's deaths were of persons under fifty years of age. This is a very serious fact which seems to suggest that nearly one half of the population lacks stamina. After making allowance for infant mortality and the fact that the whole of the still far too heavy maternal mortality is included in this figure, we are left with a situation which is thoroughly unsatisfactory. It is, no doubt, the old story of the survival of the fittest, but it is the work of sanitary science, in the widest meaning of the phrase, to increase the proportion of the fit. At present that proportion is far too small, and Sir George Newman very cogently insists upon the business value of good health. In addition to the purely human side of the matter—the misery, suffering, and sorrow produced by ill-health—he points out that "national ill-health loses time and reduces production," and that we are "in friendly competition with communities who are giving more public attention to social and health problems than we are ourselves." The sickness and disablement payments of 1923 represent a loss, through illness of greater or less severity, of more than 394,000 years. This is even worse than the loss caused by strikes, heavy as that is, and it is obvious that a nation which has to face these two enormous sources of loss is severely handicapped in the race for the supremacy of the world. The time is coming when not armaments, but health and education will ensure that supremacy, and the two necessarily run in double harness. The health side of our elementary education is, indeed, its outstanding success, since its product can neither write well nor spell well, and is abominably deficient in manners.

The moral of Sir George Newman's report and of all that we know about the health position of the country is that, while we have made a good beginning, we have a long way to go before our sanitary administration matches the progress of medicine. Opportunities and facilities are at hand, but Sir George Newman complains that in "hundreds of sanitary districts" we fail to use them, and that both local authorities and medical officers are often content to "muddle" on from hand to mouth instead of getting a comprehensive view of their work and organising it completely. It is, however, only fair to remember that the organisation of our health services is still comparatively new, and that the country has only recently begun to deal seriously with this great subject. That we have made progress the figures demonstrate, but we have still to lament a vast amount of ignorance and indifference on the side of the people and a certain amount of failure on the side of science. We have neither mastered nor even discovered, the cause of cancer or rheumatism, of measles or influenza—all of them diseases which take a heavy toll of life and, when they are not fatal, cause incalculable suffering and a vast proportion of disability. Happily we have sturdy material to work upon, and our duty now is to prevent its pitiable waste. Between unwarranted elation and the absurdities of pessimism there is a wide field for common sense and the lessons of history. As Dr. Shrubsall reminded the British Association, we have in no way deteriorated since the days of Agincourt and Waterloo. It is, indeed, probable that our powers of physical resistance are greater than ever they were, even as it is certain that our stature is greater—the war at all events proved that we are not the weaklings that our enemies believed, and that we half suspected ourselves to be. Yet the strain of modern life is heavy, and the conditions under which it is lived are often far from ideal; we crowd the towns and leave the broad pastures and the perfumed plough lands empty. Yet at the age when the second great Cecil craved for death we begin to look forward to the most fruitful, and often perhaps the happiest, period of life and activity.

